# Supplementary material for: Resolving the full spectrum of human genome variation using Linked-Reads
Source: Genome Res. 2019 Apr;29(4):635–45. doi: 10.1101/gr.234443.118 (PMC6442396; doi:10.1101/gr.234443.118)
Supplement: Supplemental Material [file supp_gr.234443.118_Supplemental_longranger-2.2.zip › longranger-2.2.tar.bz2!/lr2.2-public/lib/stan/cmdstan/stan/doc/api/html/structstan_1_1contains__fvar.html]

Stan: stan::contains\_fvar< T1, T2, T3, T4, T5, T6 > Struct Template Reference


|  |  |
| --- | --- |
|  | Stan  2.5.0  probability, sampling & optimization |


- Main Page
- Related Pages
- Namespaces
- Classes
- Files

- Class List
- Class Index
- Class Hierarchy
- Class Members

All Classes Namespaces Files Functions Variables Typedefs Enumerations Enumerator Friends Macros Pages

- stan
- contains\_fvar

Public Types |
List of all members

stan::contains\_fvar< T1, T2, T3, T4, T5, T6 > Struct Template Reference

Metaprogram to calculate the base scalar return type resulting from promoting all the scalar types of the template parameters.
More...

`#include <traits.hpp>`

|  |  |
| --- | --- |
| Public Types | |
| enum | { value } |
|  | |

## Detailed Description

### template<typename T1, typename T2 = double, typename T3 = double, typename T4 = double, typename T5 = double, typename T6 = double> struct stan::contains\_fvar< T1, T2, T3, T4, T5, T6 >

Metaprogram to calculate the base scalar return type resulting from promoting all the scalar types of the template parameters.

Definition at line 443 of file traits.hpp.

## Member Enumeration Documentation

template<typename T1 , typename T2 = double, typename T3 = double, typename T4 = double, typename T5 = double, typename T6 = double>

anonymous enum

| Enumerator | |
| --- | --- |
| *value* |  |

Definition at line 444 of file traits.hpp.

---

The documentation for this struct was generated from the following file:

- src/stan/meta/traits.hpp


---

[ Stan Home Page ]

*© 2011–2014,
Stan Development Team.*
